# Ultrafast electron diffraction from a Bi(111) surface: Impulsive lattice excitation and Debye–Waller analysis at large momentum transfer

**DOI:** 10.1063/1.5093637

**Published:** 2019-05-01

**Authors:** V. Tinnemann, C. Streubühr, B. Hafke, A. Kalus, A. Hanisch-Blicharski, M. Ligges, P. Zhou, D. von der Linde, U. Bovensiepen, M. Horn-von Hoegen

**Affiliations:** Department of Physics and Center for Nanointegration (CeNIDE), University of Duisburg-Essen, 47048 Duisburg, Germany

## Abstract

The lattice response of a Bi(111) surface upon impulsive femtosecond laser excitation is studied with time-resolved reflection high-energy electron diffraction. We employ a Debye–Waller analysis at large momentum transfer of 9.3 Å^−1^ ≤ Δ *k* ≤ 21.8 Å^−1^ in order to study the lattice excitation dynamics of the Bi surface under conditions of weak optical excitation up to 2 mJ/cm^2^ incident pump fluence. The observed time constants *τ*_int_ of decay of diffraction spot intensity depend on the momentum transfer Δ*k* and range from 5 to 12 ps. This large variation of *τ*_int_ is caused by the nonlinearity of the exponential function in the Debye–Waller factor and has to be taken into account for an intensity drop Δ*I* > 0.2. An analysis of more than 20 diffraction spots with a large variation in Δ*k* gave a consistent value for the time constant *τ_T_* of vibrational excitation of the surface lattice of 12 ± 1 ps independent on the excitation density. We found no evidence for a deviation from an isotropic Debye–Waller effect and conclude that the primary laser excitation leads to thermal lattice excitation, i.e., heating of the Bi surface.

## I. INTRODUCTION

Bismuth is a prototypical model system for studies of laser induced energy transfer from an excited electron system to the lattice system in the time domain. In its most common form, Bi exhibits the *α*-arsenic or A7 structure^1^ and is a semimetal with the conduction band slightly lower in energy than the valence band. The charge carriers are holes at T point and electrons at L point in the Brillouin zone.^2^ The almost vanishing density of states at the Fermi energy results in a low number of free carriers of 10^17^–10^19^ cm^−3^. This makes this material very sensitive to optical excitations as changes in the electron occupation affects the potential energy surface and trigger atomic motion through displacive excitation. Bismuth is subject to a Peierls distortion which breaks the translational symmetry along the (111) direction. The crystal basis consists of two Bi atoms: atom 1 on an undistorted lattice site and atom 2 at a position slightly displaced from the center along the body diagonal of the unit cell. This equilibrium structure, in particular, the distance of the two atoms of the basis, can easily be perturbed by electronic excitation.^3,4^ When the distance is changed by an ultrafast displacive excitation, the Bi atoms perform a damped oscillation along the body diagonal. This mode of coherent atomic motion represents a symmetric A_1g_ optical phonon mode of the crystal.^5–10^

Depending on the degree of fs (femtosecond)-laser optical irradiation, vastly different time constants for the excitation process of the Bi lattice were observed. Strong excitation with fluences of more than 6 mJ/cm^2^ generates so many electron hole pairs that this causes a rapid change in the potential energy surface resulting in nonthermal melting. For fluences of 18 mJ/cm^2^, the electronic acceleration of the atomic motion occurs as fast as 190 fs, resulting in ultrafast melting, destruction of the Bi-film, and a coherent A_1g_ phonon mode is not observed.^11,12^

For fluences lower than 6 mJ/cm^2^, the lattice response is reversible, the coherent A_1g_ optical phonon mode is excited,^8^ and the bond softening occurs which results in an inverse Peierls transition.^7,12–14^ Subsequently, the lattice is heated on slower time scales of 2–4 ps (Refs. 8, 11, and 15–17) through energy transfer from the electron system to the lattice by electron phonon coupling and anharmonic coupling of the A_1g_ mode to acoustic phonons.^18^ The vibrational excitation of the surface atoms is even slower: thermal motion of the Bi surface atoms sets in on a timescale of 12 ps and has been attributed to the weak coupling between bulk and surface phonons.^16^

Due to its high-atomic mass and weak bonds (the melting temperature is 271 °C), bismuth exhibits a low-Debye temperature of Θ_D_ = 112 K^19^ and thus a large vibrational amplitude of the thermal motion. These large displacements make Bi an ideal model system to study lattice dynamics upon ultrashort optical excitation by means of diffraction techniques.

Here, we present a study of the lattice response of a Bi(111) surface upon fs-laser excitation. We analyze the lattice excitation of the surface atoms through time-resolved reflection high-energy electron diffraction (RHEED). Using the Debye–Waller effect, the onset of atomic motion was directly accessible in earlier studies through the transient intensity changes in the diffraction patterns.^20–24^ Electron diffraction allows for a large momentum transfer due to the possible large scattering angles which result in large intensity changes. Employing all detected diffraction spots of the RHEED pattern for the analysis provides the variation of the momentum transfer Δ**k** of diffraction, i.e., a wide range of parallel $k_{\left|\right|}$ and vertical $k_{⊥}$ momentum transfers are available all at once. Such analysis is reported here.

The grazing incidence of the probing electrons ensures the necessary surface sensitivity and only the topmost bilayer of the Bi film contributes to the RHEED pattern.^25,26^ The excitation of the surface lattice is followed by means of the Debye–Waller effect $I/I_{0}=\text{exp}\left(-{\left\langle u\cdot \Delta k\right\rangle }^{2}\right)$ with the vibrational amplitude **u** of the atoms, the momentum transfer Δ**k**, and the stationary sample.

If the intensity drop $\Delta I(t)=1-I(t)/I_{0}$ is not too large, i.e., Δ*I*(*t*) < 0.2, then the intensity evolution *I*(*t*)/*I*_0_ can linearly be converted with an error of less than 6% in the time constant to a transient change in vibrational amplitude **u**(*t*) applying the linear expansion of the exponential function. This linear expansion, however, becomes inapplicable for intensity drops Δ*I*(*t*) > 0.2 which easily occurs for systems with a low-Debye temperature, strong excitation, or diffraction at large momentum transfer Δ*k*. Then, the intensity *I*(*t*) decays with a time constant which becomes significantly shorter with the increase in the intensity drop Δ*I*(*t*).

Here, we used RHEED spots on three different Laue circles, i.e., with different $k_{\left|\right|}$ and $k_{⊥}$, and various laser pump fluences for the excitation of the Bi(111) film in order to analyze the lattice dynamics of the Bi(111) surface. The nonlinearity of the exponential function causes the decrease in the time constant *τ*_int_ for the decay of RHEED spot intensity from 11 ps to 5 ps with the increase in the laser fluence Φ and the increase in the momentum transfer Δ**k**. Irrespective of this large variation of *τ*_int_, we obtain a time constant of 12 ps for the heating of the bismuth surface which is independent of the level of excitation.

## II. EXPERIMENTAL SETUP AND METHODS

### A. Experimental setup

**FIG. 1.**
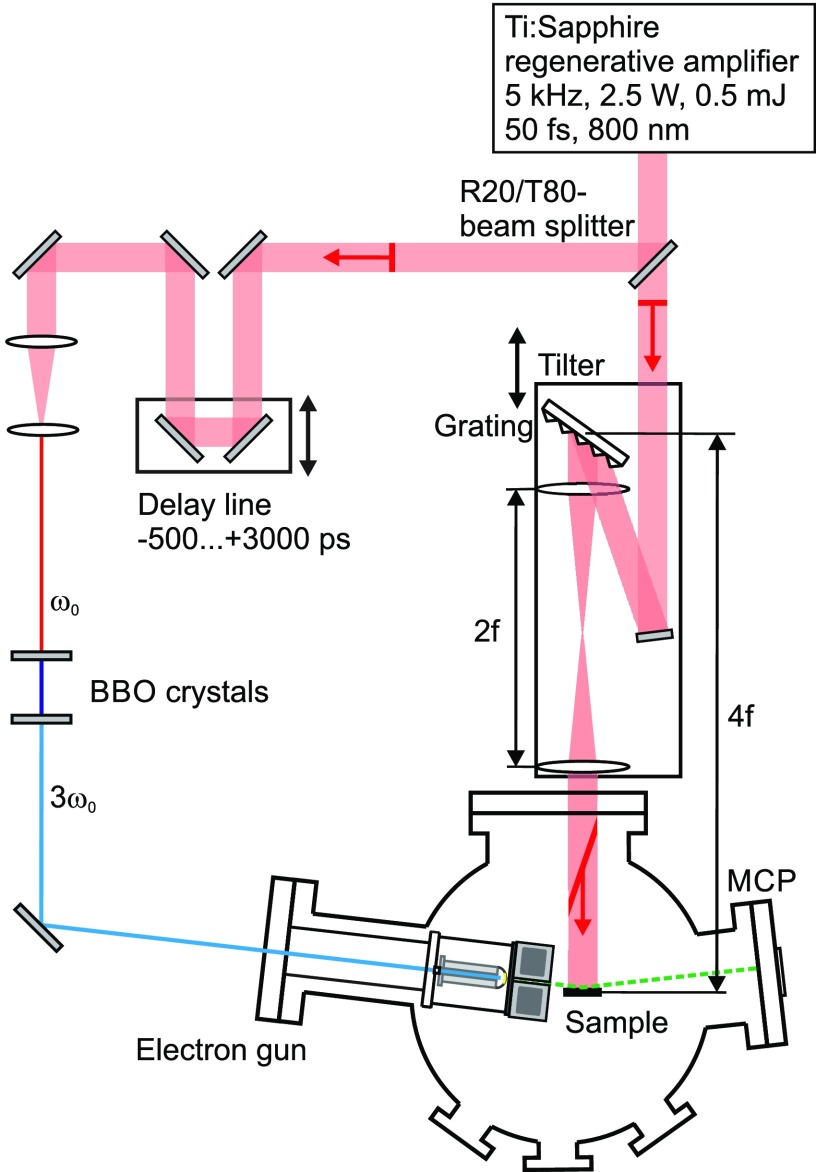
Scheme of the experimental setup. The fundamental of the laser system is split by a R20/T80 beam splitter. The 20%-part is frequency tripled and generates the probing electron pulse via photo electron emission in a back-illuminated transparent gold photo cathode. The electron pulse is accelerated to a kinetic energy of 26 keV and is diffracted at the sample under grazing incidence. The diffraction pattern is detected by an MCP amplifier with a phosphor screen and recorded by a CCD camera. The 80%-part of the initial laser pulse is used to excite the sample under normal incidence. For compensation of the velocity mismatch and to ensure—over the entire sample—temporal and spatial overlap with zero time delay of probing electrons and pumping laser pulse, the pumping laser intensity pulse front is tilted by a grating in a 4f setup. The time delay between laser pump pulse and electron probe pulse is varied by an optomechanical delay line.

The time-resolved RHEED experiments are performed under ultrahigh vacuum conditions at a base pressure below 2 × 10^−10^ mbar. A scheme of the experimental laser pump–electron probe setup is shown in Fig. 1. An amplified Ti:sapphire laser system delivers laser pulses with a central wavelength of 800 nm, a duration of 50 fs, and a pulse energy of 0.5 mJ at a repetition rate of 5 kHz. The third harmonic of the fundamental generates electron pulses via photo electron emission in a back-illuminated transparent gold cathode.^27^ The electrons are accelerated to 26 keV with a de Broglie wavelength of *λ* = 7.6 pm or momentum *k*_0_ = 2*π*/*λ* = 82.6 Å^−1^ and are diffracted at the sample under a grazing incidence of 3.4°, i.e., resulting in a vertical momentum transfer of 9.3 Å^−1^ for the specular (00)-spot. The diffraction pattern is intensity amplified by a microchannel plate, detected by a phosphor screen, and recorded by a cooled CCD camera. The sample is excited by 800 nm laser pulses under normal incidence at pump powers up to 1200 mW, corresponding to an incidence fluence of Φ = 2 mJ/cm^2^, adjusted by a combination of half-wave plate and thin film polarizer.^28^ The incidence fluence is determined with a systematic error of −30% +40%. To record the dynamics after laser excitation, the time delay between pumping laser pulse and probing electron pulse is varied by an optomechanical delay line. The grazing incidence of the electrons leads to a systematic change in the arrival times of the electrons across the sample. This so-called velocity mismatch of pumping laser pulse and probing electron pulse limits the temporal resolution to a few 10 ps.^29^ To compensate this effect and to ensure constant time delays across the sample, the pumping laser pulse intensity front is tilted^30^ by an angle of 71° as described in detail in Ref. 31.

Two different sample systems were *in-situ* prepared and studied: few nm thick epitaxial Pb islands on Si(111) and a 8 nm thin epitaxial Bi(111) film grown on a clean Si(111)–(7 × 7) reconstructed substrate.^32,33^ The Pb islands are prepared by the deposition of Pb on a Si(111)–(7 × 7) reconstructed substrate at 300 K. Pb is known to have a large electron phonon coupling constant^34^ and the lattice response time after laser excitation is thus expected to be fast. Pb islands on Si(111) were therefore used to determine an upper limit for the temporal instrumental response function of the experiment. In contrast, Bi exhibits an electron phonon coupling that is much weaker^35^ compared to Pb and therefore the response time is expected to be slower than the temporal resolution of the experiment.

### B. Data analysis

**FIG. 2.**
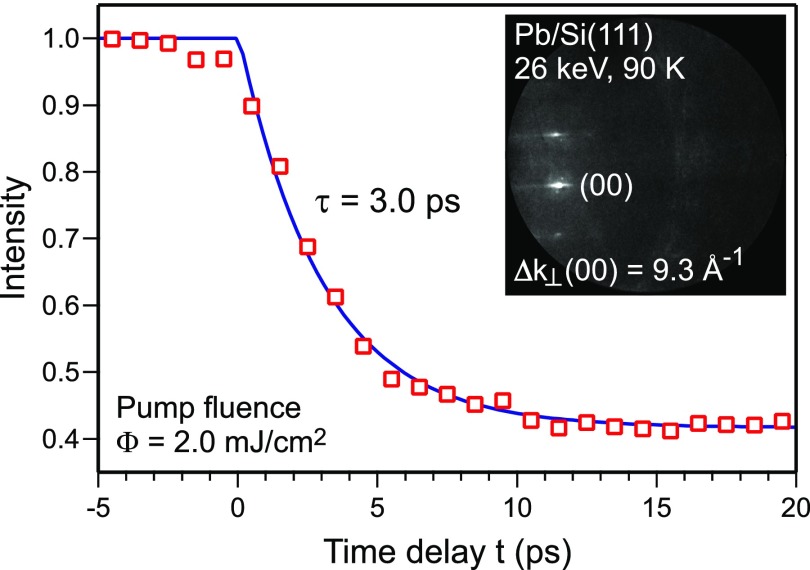
Impulsive heating of epitaxial lead islands on Si(111) at *T*_0_ = 90 K. The normalized intensity of the (00)-spot is plotted as function of the time delay between pump and probe pulse. The data are fitted with an exponential decay function (solid line). The time constant *τ_int_* was found to be 3.0 ps. The diffraction pattern was taken at an electron energy of 26 keV with a grazing angle of incidence of 3.4°.

The intensity of the diffraction spots was determined from a line profile through the spots. The profile was fitted with a Gaussian function and the value for the absolute intensity is given by the maximum of the Gaussian fit. The intensity is normalized to the intensity prior to excitation at a sample temperature of *T*_0_ = 90 K. In Fig. 2, this intensity $I(t)/I_{T_{0}}$ of the (00)-spot of Pb islands on Si(111) is plotted as a function of the delay between pump and probe pulse. For negative time delays, the intensity remains constant: the surface is probed before excitation. At the temporal overlap of pump and probe pulse, the intensity decreases as caused by the Debye–Waller effect. Subsequent to the laser irradiation, the lattice system is excited, the vibrational amplitude **u** of the surface atoms increases, and the spot intensity is reduced.^36,37^ The intensity as a function of the change in vibrational amplitude $\Delta u(t)=u(t)-u_{T_{0}}$ can be described by

$$I(t)/I_{T_{0}}=\;\text{exp}\left(-{\left\langle \Delta u(t)\cdot \Delta k\right\rangle }^{2}\right),$$ (1)

which, for an isotropic vibrational amplitude, is simplified to

$$\begin{array}{c} I(t)/I_{T_{0}}=\text{exp}\left(-1/3\;\Delta \langle u{\left(t\right)}^{2}\rangle \Delta k^{2}\right) \end{array},$$ (2)

with $\Delta \langle u{\left(t\right)}^{2}\rangle$ being the transient change in the mean squared displacement (MSD). The measured transient intensity decay is typically fitted by an exponential function

$$\frac{I(t)}{I_{90\;K}}=1-\Delta I_{\text{max}}\cdot \Theta (t)\left(1-\text{exp}\left(-t/\tau_{\text{int}}\right)\right),$$ (3)

with the Heaviside step function Θ(*t*), the maximum intensity drop Δ*I*_max_, and the decay time constant *τ*_int_ for the intensity. The experimental decay time constant for the intensity of the (00)-spot of the Pb(111) islands on Si(111) is *τ*_int_ = 3.0 ± 0.4 ps. The fit is shown as a solid line. Thus, the temporal resolution of the time resolved RHEED experiment at 26 keV is better than 3 ps.

### C. Diffraction geometry

**FIG. 3.**
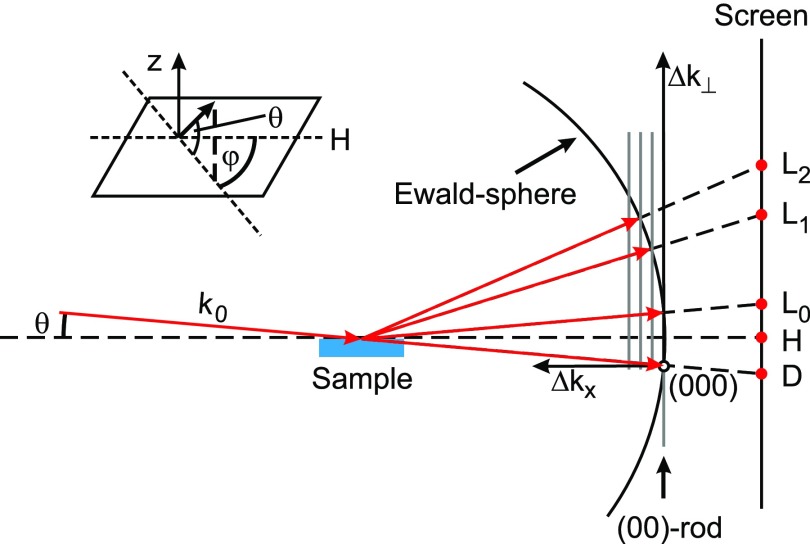
Scheme of momentum transfer in RHEED adopted from Ref. 53. The incident electrons have a momentum $k_{0}$ and are diffracted at the surface of the sample. The origin of reciprocal space is marked by (000). The position of the diffraction spots on the screen is determined by the projection of the intersection of the lattice rods with the Ewald sphere. *L_n_* indicates the different orders of Laue circles.

The Debye–Waller effect depends on the vector of momentum transfer $\Delta k_{k,l}$ of the specific diffraction spot (*k*,*l*). Therefore, the precise knowledge of the diffraction geometry and the resulting momentum transfers $\Delta k_{k,l}$ of diffraction spots of order (*k*,*l*) is indispensable. A scheme of the diffraction geometry in RHEED is shown in Fig. 3. The incident electrons with an initial momentum of $k_{0}$ define the radius of the Ewald sphere and the origin of the reciprocal lattice. The diffracted electrons have undergone a momentum transfer of $\Delta k_{k,l}=k_{0}-k_{k,l}$ which can be separated into a component normal to the surface ($\Delta k_{k,l,⊥}$) and components parallel to the surface ($\Delta k_{k,l,x}$ and $\Delta k_{k,l,y}$). For the 0th order Laue circle (*L*_0_), the momentum transfer in x-direction is zero and increases by one parallel reciprocal lattice distance from Laue circle to Laue circle. The second component parallel to the surface Δ*k_y_* is oriented normal to the plane spanned by the initial and specular beams.

**FIG. 4.**
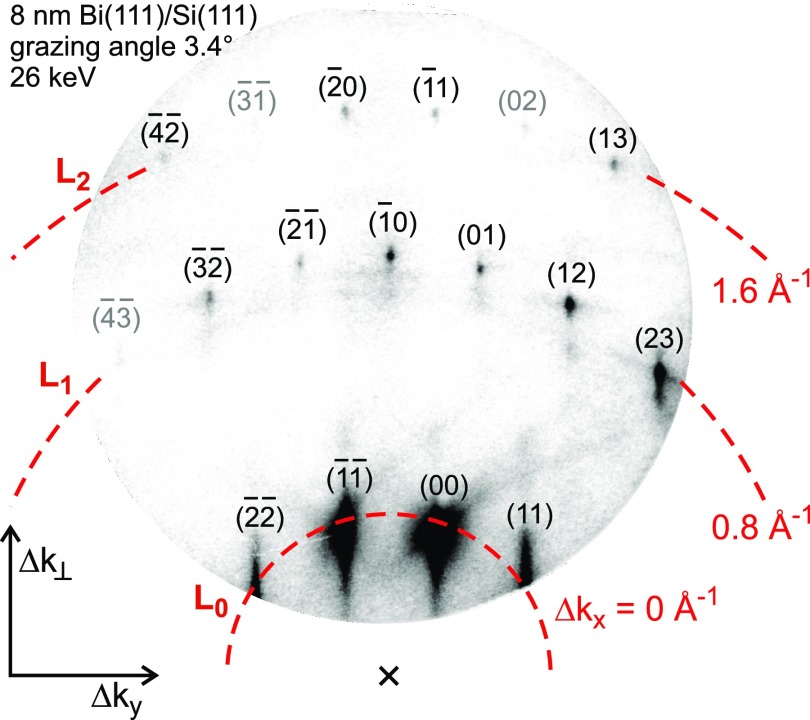
Diffraction pattern of Bi/Si(111) recorded at an energy of 26 keV and a sample temperature of *T*_0_ = 90 K. The grazing angle of incidence of the electrons was 3.4°. The vertical $\Delta k_{⊥}$ and parallel $\Delta k_{y}$ momentum transfer of the diffracted electrons are indicated. The momentum transfer in x-direction (along the incident electron beam, see Fig. 3) depends on the order of Laue circle (dashed lines indexed by L_0_, L_1_, and L_2_).

Figure 4 shows the diffraction pattern of the Bi(111)-film grown on Si(111) taken at an electron energy of 26 keV, a grazing angle of incidence of 3.4°, and a static sample temperature of 90 K. The momentum transfer is determined for all diffraction spots from diffraction geometry and reciprocal lattice constants. The diffraction pattern is shown in units of $\Delta k_{⊥}$ (left axis) and Δ*k_y_* (bottom axis). Δ*k_x_* increases with the order of Laue circles (dashed lines). Here, the (00)-rod is not in the center of the Laue circle because the incident electrons exhibit an azimuth angle *φ* of 1° from the [112] direction. The values for $\Delta k_{⊥}$ cover the range from 7 to 22 Å^−1^. The momentum transfer $\left|\Delta k_{\left|\right|}\right|$ parallel to the surface is below 8 Å^−1^ for all observed spots. Since $\Delta k_{⊥}\gg \left|\Delta k_{\left|\right|}\right|$, our experiment is mainly sensitive to a change in the vibrational amplitude perpendicular to the surface.

## III. RESULTS AND DISCUSSION

**FIG. 5.**
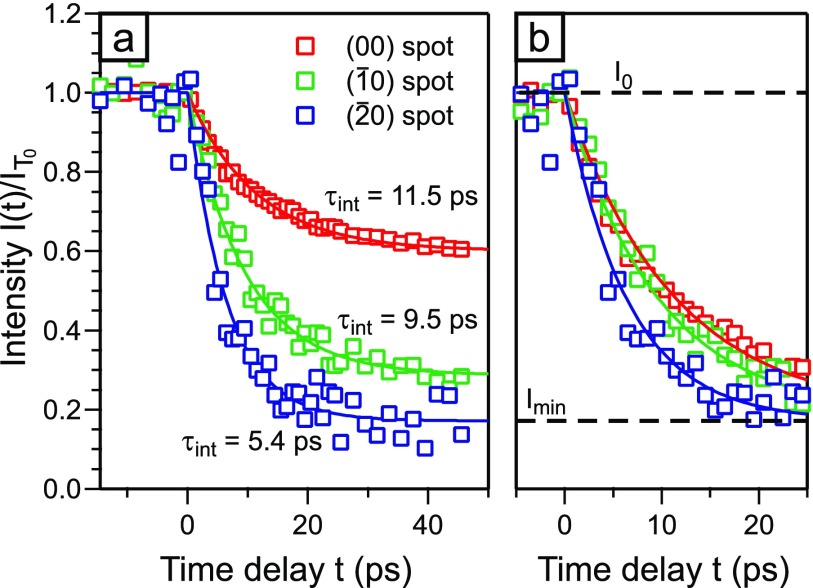
(a) The intensity $I(t)/I_{T_{0}}$ as a function of the time delay is shown for three diffraction spots on different Laue circles (red: 0th, green: 1st, and blue: 2nd). The intensity drop Δ*I*_max_ increases with momentum transfer from 40% to more than 80%, while the time constant decreases from 11.5 ps to 5.4 ps. (b) The intensity is normalized to the intensity drop to illustrate the difference in the time constants *τ*_int._ The incident pump laser fluence is Φ ≃ 1.4 mJ/cm^2^.

Time-resolved RHEED measurements were performed on an 8 nm thin Bi-film on Si(111). The base temperature of the sample was *T*_0_ = 90 K and the incident pump laser fluence Φ ≃ 1.4 mJ/cm^2^. The intensity of all diffraction spots is analyzed as a function of time delay. In Fig. 5(a), the intensity evolution is exemplarily shown for diffraction spots on the three Laue circles: the (00)-spot, the $\left(\bar{1}0\right)$-spot, and the $\left(\bar{2}0\right)$-spot. All diffraction spots show an intensity drop that can be described by an exponential decay function. The intensity drop is caused by the Debye–Waller effect. The minimum intensity is reached after ∼40 ps. Cooling of the thin film occurs via thermal transport to the Si substrate on a timescale of 500–1000 ps^23,28^ and therefore cannot be observed on the timescale of 50 ps after excitation. The intensity decay $I(t)/I_{T_{0}}$ of the three diffraction spots in Fig. 5(a) scales with the squared momentum transfer that rises from 86.5 Å^−2^ for the (00)-spot to 472 Å^−2^ for the $\left(\bar{2}0\right)$-spot. The time constant obtained from the exponential fit decreases from 11.5 ps for the (00)-spot to 5.4 ps for the $\left(\bar{2}0\right)$-spot. To clearly illustrate the difference of the time constants, the normalized intensity drop Δ*I*(*t*) is plotted in Fig. 5(b).

In earlier works, the transient intensity of ultrathin hetero films was directly converted into a temperature curve employing a stationary calibration measurement.^23,27,28^ Here, we analyze the transient spot intensity without such conversion. For simplicity, we apply the Debye model in the high-temperature regime ($T≳\Theta_{D,\text{surf}}$) and assume an isotropic MSD $\left\langle u^{2}\right\rangle$ proportional to the temperature

$$\langle u^{2}\rangle =\frac{3\hbar^{2}T}{Mk_{B}\Theta_{D,\text{surf}}^{2}},$$ (4)

where $\Theta_{D,\text{surf}}$ is the effective surface Debye temperature in the framework of individual harmonic oscillators [$\Theta_{D,\text{surf}}=47\;K$ for the Bi(111) surface^23,38,39^] and *M* is the atomic mass of Bi. We also assume an exponential increase in MSD, i.e., an exponential rise in temperature *T*(*t*) to a maximum temperature $T_{0}+\Delta T_{\text{max}}$, with a time constant *τ_T_*,

$$T(t)=T_{0}+\Delta T_{\text{max}}\cdot \Theta (t)\left(1-\text{exp}\left(-t/\tau_{T}\right)\right).$$ (5)

The intensity is

$$\begin{array}{c} I(t)/I_{T_{0}}=\text{exp}\left[-\alpha \Delta T_{\text{max}}\cdot \Theta (t)\left(1-\text{exp}\left(-t/\tau_{T}\right)\right)\right], \end{array}$$ (6)

with $\alpha =\hbar^{2}\Delta k^{2}/Mk_{B}\Theta_{D,\text{surf}}^{2}$. For small values of $\alpha \Delta T_{\text{max}}$, we can safely use a linear approximation of the exponential because the higher order terms in the expansion are negligibly small,

$$I(t)/I_{T_{0}}≃1-\alpha \Delta T_{\text{max}}\cdot \Theta (t)\left(1-\text{exp}\left(-t/\tau_{T}\right)\right).$$ (7)

With this approximation, the maximum intensity drop is $\Delta I_{\text{max}}=\alpha \Delta T_{\text{max}}$ and the time constant *τ*_int_—as experimentally determined from the transient intensity decay—is almost the same as *τ_T_* from the temperature curve. The question arises up to what arguments *α*Δ*T*_max_ we can use the linear approximation?

**FIG. 6.**
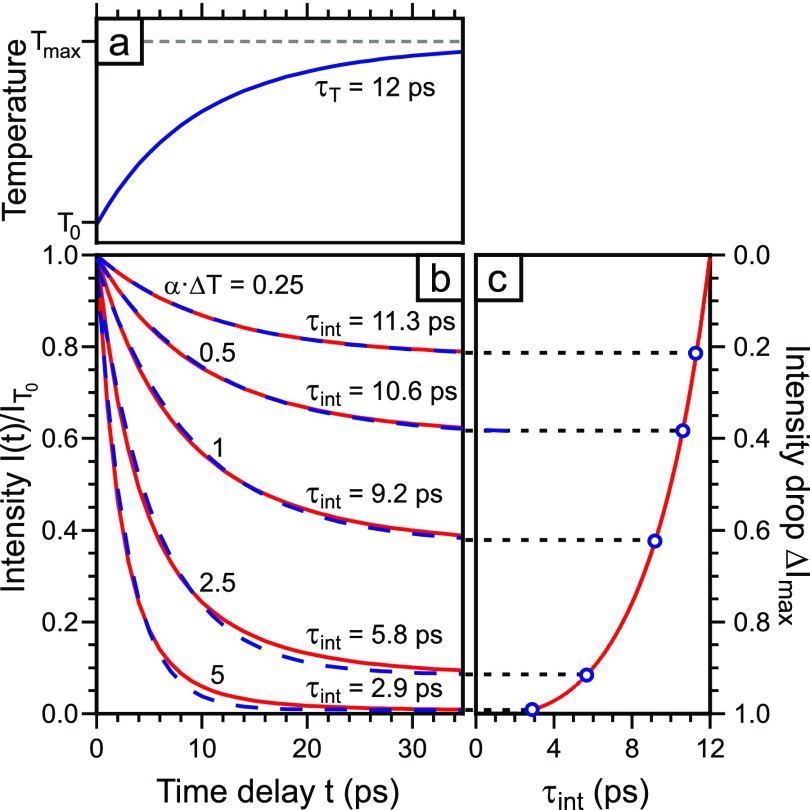
(a) Exponential temperature rise by Δ*T*_max_ with time constant *τ_T_* = 12 ps. (b) The intensity for the temperature rise in (a) is plotted as a function of the time delay with different values of *α*Δ*T*_max_ (solid lines). The curves are fitted by an exponential decay function (dashed lines). (c) The time constant obtained from the fit is plotted as a function of the intensity drop Δ*I*_max_. With the increase in the values for *α*Δ*T*_max_, the intensity drop becomes larger and the fitted time constants decrease dramatically from 12 ps for $\alpha \Delta T_{\text{max}}\approx 0$ to 2.9 ps for $\alpha \Delta T_{\text{max}}=5$.

We modeled the intensity to obtain the time constant *τ*_int_ in dependence of the intensity drop Δ*I*_max_. An exponential temperature rise with a time constant of *τ_T_* = 12 ps [see Fig. 6(a) and observed in Ref. 16] is converted into the corresponding intensity *I*(*t*) using Eq. (6). *I*(*t*) is plotted in Fig. 6(b) as a function of the time delay for 5 different values of *α*Δ*T*_max_ (solid lines) and fitted with an exponential decay function as given by Eq. (3) (dashed lines). For small values *α*Δ*T*_max_ = 0.2, the calculated intensity *I*(*t*) exhibits almost the same behavior like *T*(*t*) and is well described by the fit function [Eq. (3)]. The intensity drop Δ*I*_max_ is ≤ 18% and the time constant obtained from the exponential fit (dashed line) *τ*_int_ = 11.3 ps deviates only by 6% from *τ_T_*.

With the increase in the values for *α*Δ*T*_max_, however, the time constant obtained from the exponential fit *τ*_int_ (dashed lines in Fig. 6) decreases. In the right panel of Fig. 6, the fitted time constant *τ*_int_ is plotted as a function of the intensity drop Δ*I*_max_. For Δ*I*_max_ approaching unity, i.e., drop to intensity to almost zero, the time constant *τ*_int_ decreases to 3 ps and less. We therefore have to expect strongly varying experimental time constants *τ*_int_ depending on the degree of excitation (Δ*T*_max_) or momentum transfer (*α*). The varying time constants of 5.4–11.5 ps obtained for the different orders of Laue circles shown in Fig. 5 are thus explained by the correlation of Δ*I*_max_ and *τ*_int_ as shown in Fig. 6(c). The correct time constant of the temperature rise *τ_T_* can only be found by extrapolation to Δ*I*_max_ = 0. Therefore, under our diffraction conditions at large momentum transfer Δ*k* and large intensity drop Δ*I*_max_, the time constants *τ*_int_ can be much shorter than *τ_T_*. In the following, we perform a thorough Debye–Waller analysis in order to prove that the preconditions for such analysis are still valid.

From the change in the spot intensity, we obtain information about the change in the MSD,

$$-\text{ln}(I(T)/I_{T_{0}})=1/3\Delta k^{2}\left(\left\langle u^{2}(T)\rangle -\langle u_{T_{0}}^{2}\right\rangle \right).$$ (8)

**FIG. 7.**
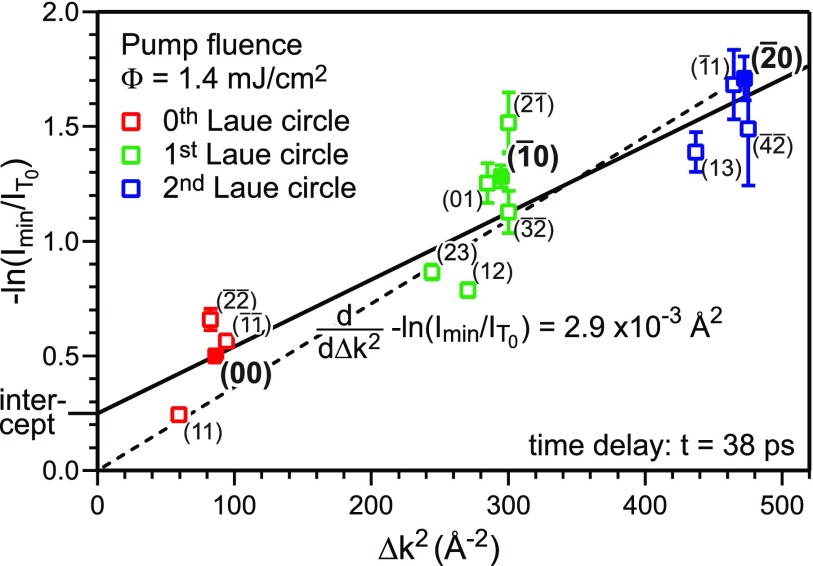
The negative logarithm of the minimum intensity $I(T_{\text{max}})/I_{T_{0}}$ is plotted as a function of Δ*k*^2^ for all diffraction spots at a time delay of *t* = 38 ps. Data from the different Laue circles are plotted in different colors. Solid data points are from spots analyzed in Fig. 5(b). If applying kinematic scattering theory, a linear fit through the origin is expected (dashed line). The solid line gives a better fit to the data and the intercept is explained by multiple scattering effects, following the literature.^40–42^

From the kinematic diffraction theory,^36,37^ we expect a linear dependence of the negative logarithm of the intensity $-\text{ln}(I(T)/I_{T_{0}})$ as a function of Δ*k*^2^ with a y-axis intercept equal to zero [Eq. (8)]. The slope $-d(\text{ln}(I(T)/I_{T_{0}}))/d(\Delta k^{2})$ is equal to one third of the change in the MSD $\Delta \langle u^{2}\rangle =\langle u^{2}(T_{\text{max}})\rangle -\langle u_{T_{0}}^{2}\rangle$ or, if the effective surface Debye temperature $\Theta_{D,\text{surf}}$ is known (here $\Theta_{D,\text{surf}}=47\;K)$, proportional to the temperature rise Δ*T*_max_, respectively. Figure 7 depicts $-\text{ln}\left(I_{\text{min}}/I_{T_{0}}\right)$ for all diffraction spots plotted as a function of the squared momentum transfer Δ*k*^2^. The value *I*_min_ is the minimum intensity obtained from the fit for the maximum transient temperature. The expected behavior for kinematic diffraction theory and isotropic vibrational motion is plotted as the dashed line. The data are, however, better described by a linear fit with a y-axis intercept > 0. Such positive intercept was also observed in transmission electron diffraction experiments^40–42^ and is caused by multiple scattering effects. The offset observed in transmission electron diffraction was found to be proportional to the temperature change as well and is explained by dynamical two beam diffraction theory.

The scatter of the data in Fig. 7 and the large variation of the intensity of the diffraction spots in Fig. 4 are attributed to multiple scattering effects. Through the fitting of the Debye–Waller drop for all the 14 analyzed diffraction spots, many of these dynamic scattering effects are averaged out and we can apply a kinematic analysis of the Debye–Waller effect. We also did not find any systematic deviations as a function of parallel $\Delta k_{\left|\right|}$ or vertical $\Delta k_{⊥}$ momentum transfer. This justifies the pre-assumption of an isotropic thermal motion. The present data do not provide insight into any potential non-equipartition in parallel or vertical vibrational amplitude. Finally, we obtain a change in the MSD at *t* = 38 ps that is $\Delta \langle u^{2}\rangle =8.8\times 10^{-3}\;{Å}^{2}$.

**FIG. 8.**
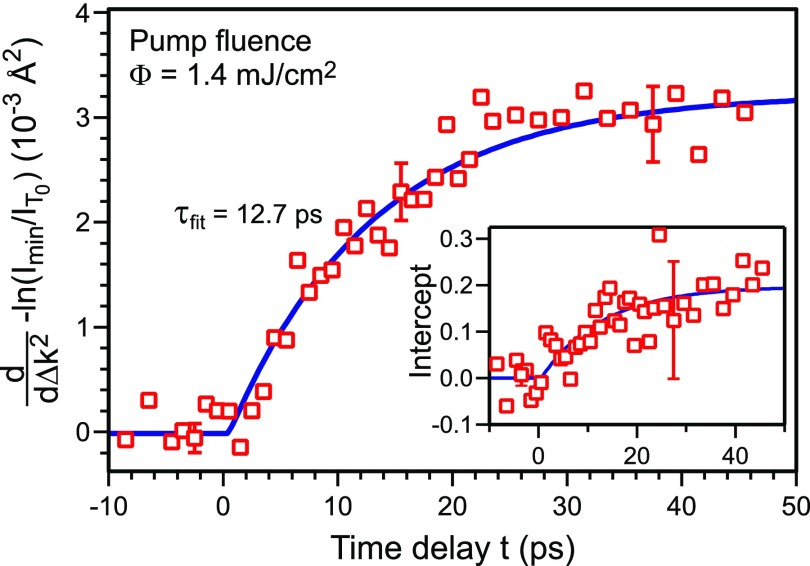
$-\text{ln}(I(T(t))/I_{T_{0}})$ as a function of $\Delta k^{2}$ was determined for all diffraction spots for each time delay step. The slope $-d(\text{ln}\;(I(T)/I_{T_{0}}))/d(\Delta k^{2})$ and intercept (inset) found from the linear fit for each time step are plotted as a function of the time delay. The data are fitted exponentially. The time constant obtained from the fit of the slope is (12.7 ± 1.3) ps. The data points of the intercept have a larger error bar, but can also be described by an exponential decay function with a fixed time constant of 12.7 ps.

To obtain information about the change in MSD $\Delta \langle u{\left(t\right)}^{2}\rangle$ as a function of time, we performed a Debye–Waller analysis same as in Fig. 7 for every time delay step during a measurement. In Fig. 8, $\Delta \langle u{\left(t\right)}^{2}\rangle$ and the intercept (inset) are plotted as function of the time delay. The change in MSD $\Delta \langle u{\left(t\right)}^{2}\rangle$ is a measure for the transient temperature *T*(*t*) and is fitted by an exponential function with a time constant of (12.7 ± 1.3) ps. Due to the noise and large error bars the intercept as a function of time was fitted with this fixed time constant of 12.7 ps (inset of Fig. 8). With the surface Debye temperature $\Theta_{D,\text{surf}}=47\;K$,^38,39,43^ the maximum change in MSD for t→∞ is converted to an asymptotic temperature change in Δ*T*_max_ = 54 K.

### A. Variation of pump fluence

**FIG. 9.**
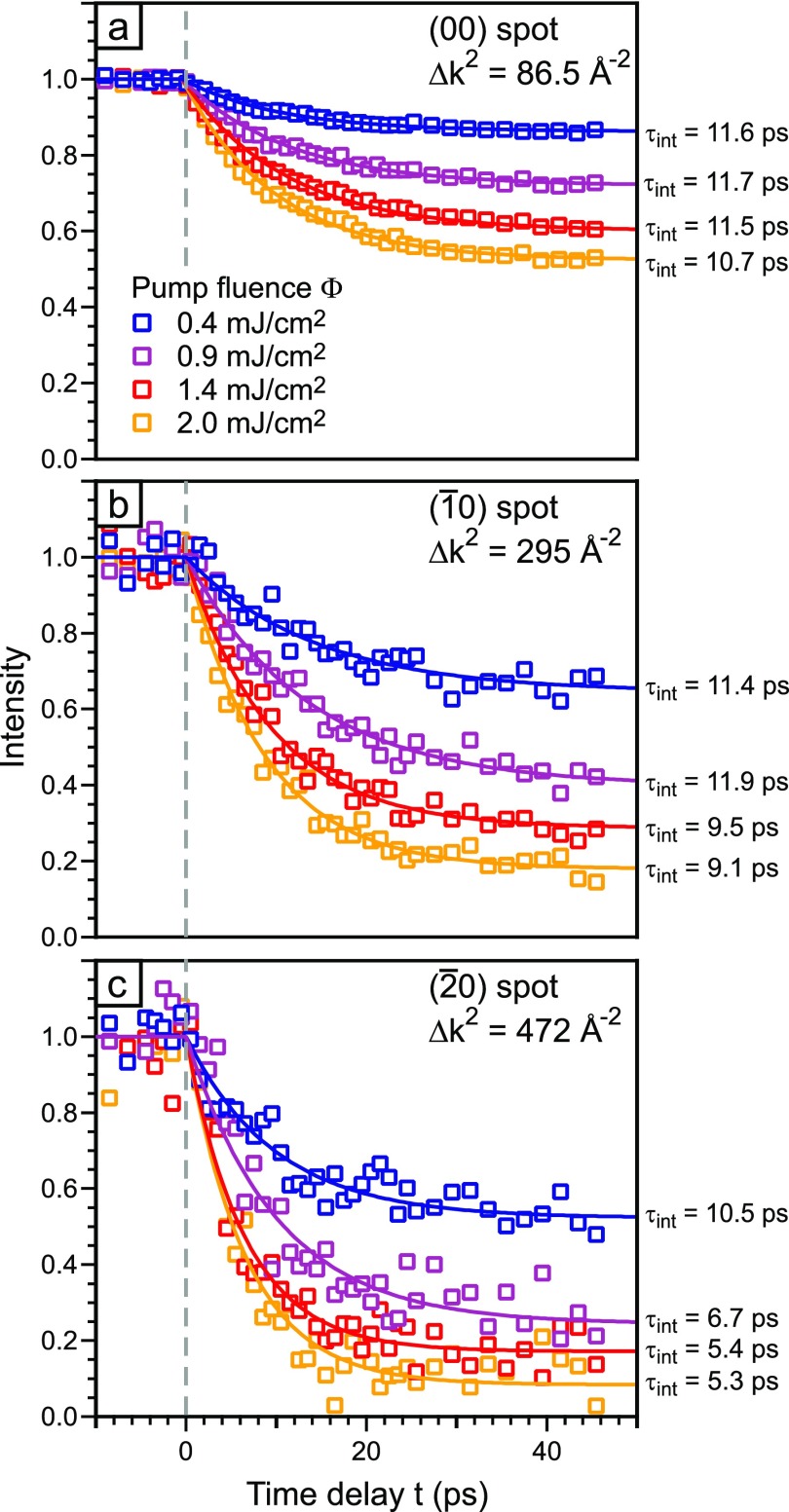
Time resolved measurements were performed with different incident pump fluences between 0.4 and 2 mJ/cm^2^. The intensity is plotted as a function of the time delay for three diffraction spots. The momentum transfer increases from (a) to (c). Data are fitted with an exponential decay function (solid lines).

The intensity drop Δ*I*_max_ depends on the absorbed energy that was changed by varying the pump fluence. In Fig. 9, the intensity as a function of the time delay is plotted for three diffraction spots (same as in Fig. 5) and four different pump fluences Φ between 0.4 and 2 mJ/cm^2^. The intensity drop Δ*I*_max_ becomes larger with the increase in the pump fluence for all diffraction spots. The (00)-spot (Δ*k*^2^ =86.5 Å^−2^) shows only a weak variation of *τ*_int_ from 11.7 to 10.7 ps upon increasing pump power. The intensity decay is still in the regime of the linear approximation and the maximum error of the time constant is <10%. For the $\left(\bar{1}0\right)$-spot (Δ*k*^2^ = 295 Å^−2^), a significant reduction of the time constant *τ*_int_ to 9.1 ps can already be observed. The $\left(\bar{2}0\right)$-spot (Δ*k*^2^ = 472 Å^−2^) exhibits intensity drops Δ*I*_max_ up to 90% and *τ*_int_ becomes shorter by a factor of more than 2 for the highest pump fluence of 2 mJ/cm^2^: a time constant of *τ*_int_ = 5.3 ps is observed.

**FIG. 10.**
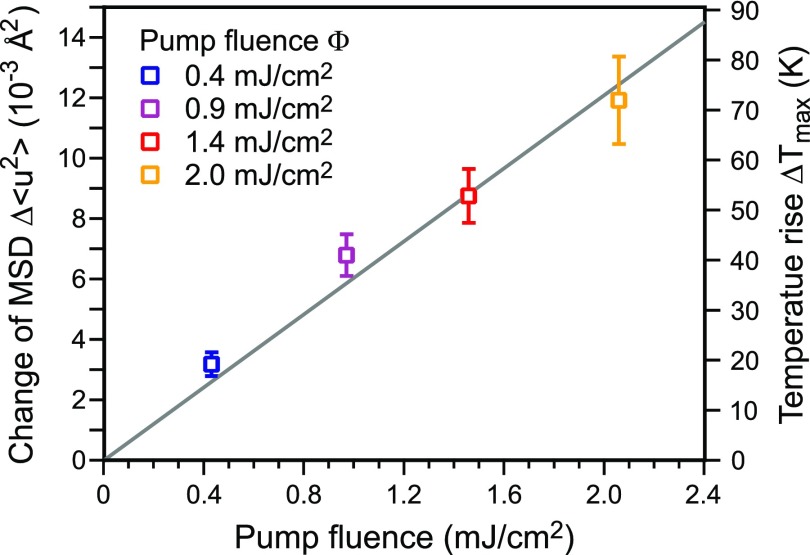
For each pump power, the slope_*DW*_ of $-\text{ln}(I(T_{\text{max}})/I_{T_{0}})$ over Δ*k*^2^ was determined same as in Fig. 7. The slope rises linear with the pump power. Thus, the change in the mean square displacement $\left\langle u^{2}\right\rangle$ as well as the change in temperature rises linear with the fluence.

For each pump fluence, a Debye–Waller analysis same as in Fig. 7 was performed for the minimum intensity *I*(*T*_max_) obtained from the exponential fit. The slope of the Debye–Waller analysis averaged over all spots is plotted as a function of pump fluence in Fig. 10. The slope and therefore the change in the MSD $\Delta \langle u^{2}\rangle$ rise linear with the pump fluence. From this, we conclude that the absorbed energy is proportional to the pump fluence and the vibrational motion of the atoms is still in the harmonic regime of the potential. For the maximum laser pump fluence of Φ = 2 mJ/cm^2^, the MSD increases by $\Delta \langle u^{2}\rangle =11.9\times 10^{-3}\;{Å}^{2}$. This corresponds to an asymptotic temperature rise in Δ*T*_max_ = 72 K.

### B. Comparison of experiment and modeling

**FIG. 11.**
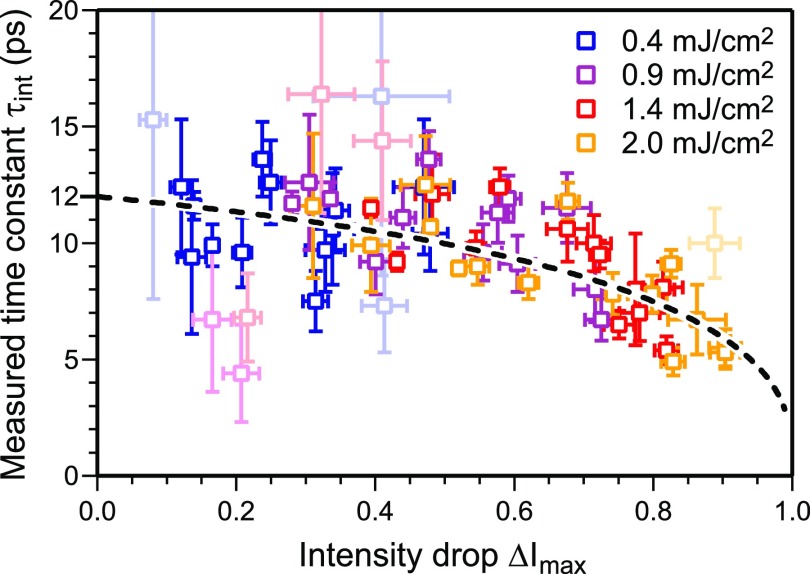
The measured time constant *τ_int_* is plotted versus the intensity drop Δ*I* for all diffraction spots and all pump fluences. The light symbols represent weak diffraction spots with strong noise and large error bars. The dashed line is the expected behavior for a temperature rise Δ*T* with a time constant of 12 ps.

Increasing pump fluence and analysis of spots with larger momentum transfer Δ*k* result in the increase in the intensity drops Δ*I*_max_ and thus shorter time constants *τ_int_*. The modeling shown in Fig. 6 explains this correlation well. Figure 11 summarizes all the experimental results and compares them with the expected behavior of *τ*_int_ (Δ*I*) shown in Fig. 6 (dashed line). For each diffraction spot, the time constant determined from the fit is plotted over the intensity drop for all four pump fluences. Light symbols represent weak diffraction spots exhibiting strong noise. For the determination of the time constant *τ_T_* of the temperature rise, we modeled $\tau_{\text{int}}(\Delta I_{\text{max}})$-curves for different values of *τ_T_* and found a minimum standard deviation for *τ_T_* = (12.0 ± 0.4) ps. No evidence for a dependence of excitation time constant *τ_T_* on the excitation level is found in the regime of weak excitation with incident pump fluences Φ ≤ 2 mJ/cm^2^.

## IV. CONCLUSION

We determined the thermal response time of the surface atoms of a Bi(111)-film upon fs-laser excitation with ultrafast time resolved RHEED. The Debye–Waller effect $I(t)/I_{0}=\text{exp}\left(-1/3\langle u{\left(t\right)}^{2}\rangle \Delta k^{2}\right)$ was employed to follow the onset of vibrational motion *u*(*t*) of the surface atoms. The measured time constant *τ*_int_ of the decay of the diffraction spot intensity *I*(*t*) varied from 5.3 to 11.7 ps and was found to be strongly dependent on the relative intensity drop $\Delta I_{\text{max}}$. Thus, *τ*_int_ depends both on the temperature rise Δ*T*_max_ and the momentum transfer Δ*k* of the specific diffraction spot under investigation. For large intensity drops Δ*I*_max_ > 0.2, the nonlinearity of the exponential function has to be considered as it results in faster intensity decays and seemingly faster time constants. This situation easily occurs for systems with a low-Debye temperature and strong excitation and/or large momentum transfer Δ*k* during diffraction.

Taking care for the aforementioned effect, we found a constant value for the time constant for the rise in Bi surface temperature of *τ_T_* = 12 ps independent of the excitation level, i.e., variation of temperature rise Δ*T*_max_ between 18 and 72 K. Almost, the same time constant of 12.7 ps is observed for the analysis of the transient change in the mean square displacement $\left\langle u{\left(t\right)}^{2}\right\rangle$ which is a direct measure of temperature, too. We thus observe a time constant for heating of the surface atoms which is more than 4 times larger than values reported for the bulk under conditions comparable to our incident laser fluences.^16^ Thus, the surface is not following the excitation of the bulk. Instead, the thermal excitation of the surface atoms occurs delayed on a time scale of 12 ps.

These findings can be explained within two different scenarios: In the first scenario, we attribute the slow excitation to a reduced electron phonon coupling at the surface. The Bi(111) surface exhibits a pronounced electronic surface state.^35,44–48^ This surface state is easily populated upon fs IR irradiation.^49^ The number of excited electrons in this surface state exhibits a lifetime comparable to the thermalization time constant observed in our experiment [see Fig. 5(a) of Ref. 49]. Both time scales for the de-excitation of the electron system and for the rise in vibrational amplitude of the lattice system are almost identical. It may thus be plausible that weak electron–phonon coupling in the surface state directly excites phonon modes at the surface.

The second scenario relies on a mechanism as it was proposed by Waldecker *et al.*: Photoexcitation generates surface and bulk carriers in the film. Through electron phonon coupling in the bulk primarily, a nonequilibrium population of optical phonons in the film is generated. This initial excitation of high-frequency optical phonons anharmonically decays into thermalized acoustic phonons on a ∼10 ps timescale.^50,51^ Because optical phonons at the same energy density exhibit a smaller vibrational amplitude than low-frequency acoustic phonons, we would also expect a delayed drop of intensity as the Debye–Waller effect is sensitive to $\left\langle u^{2}\right\rangle$ and thus less sensitive to optical phonons.^52^ Variations of the strength of anharmonic coupling by changes in the sample temperature or manipulation of the electronic surface state are promising routes for future clarification of the mechanism of lattice excitation.
